# Difference in Step-Wise Production Rules of SP Binary Flooding for Conglomerate Reservoirs with Different Lithologies

**DOI:** 10.3390/polym15143119

**Published:** 2023-07-21

**Authors:** Jianrong Lv, Guangzhi Liao, Chunmiao Ma, Meng Du, Xiaoguang Wang, Fengqi Tan

**Affiliations:** 1 Institute of Porous Flow and Fluid Mechanics, Chinese Academy of Sciences, Langfang 065007, China; 2 Xinjiang Oilfield Company, PetroChina, Karamay 834000, China; 3 Oil, Gas and New Energies Company, PetroChina, Beijing 100007, China; 4 College of Earth and Planetary Sciences, University of Chinese Academy of Sciences, Beijing 100049, China

**Keywords:** conglomerate reservoir, surfactant–polymer binary flooding, anisotropy, step-wise production, enhanced oil recovery

## Abstract

The purpose of this study is to clarify the difference in oil production rules of conglomerate reservoirs with different pore structures during surfactant–polymer (SP) binary flooding and to ensure the efficient development of conglomerate reservoirs. In this paper, the full-diameter natural cores from the conglomerate reservoir of the Triassic Kexia Formation in the seventh middle block of the Karamay Oilfield (Xinjiang, China) are selected as the research objects. Two schemes of single constant viscosity (SCV) and echelon viscosity reducing (EVR) are designed to displace oil from three main oil-bearing lithologies, namely fine conglomerate, glutenite, and sandstone. Through comprehensive analysis of parameters, such as oil recovery rate, water content, and injection pressure difference, the influence of lithology on the enhanced oil recovery (EOR) of the EVR scheme is determined, which in turn reveals the differences in the step-wise oil production rules of the three lithologies. The experimental results show that for the three lithological reservoirs, the oil displacement effect of the EVR scheme is better than that of the SCV scheme, and the differences in recovery rates between the two schemes are 9.91% for the fine conglomerate, 6.77% for glutenite, and 6.69% for sandstone. By reducing the molecular weight and viscosity of the SP binary system, the SCV scheme achieves the reconstruction of the pressure field and the redistribution of seepage paths of chemical micelles with different sizes, thus, achieving the step-wise production of crude oil in different scale pore throats and enhancing the overall recovery of the reservoir. The sedimentary environment and diagenesis of the three types of lithologies differ greatly, resulting in diverse microscopic pore structures and differential seepage paths and displace rules of SP binary solutions, ultimately leading to large differences in the enhanced oil recoveries of different lithologies. The fine conglomerate reservoir has the strongest anisotropy, the worst pore throat connectivity, and the lowest water flooding recovery rate. Since the fine conglomerate reservoir has the strongest anisotropy, the worst pore throats connectivity, and the lowest water flooding recovery, the EVR scheme shows a good “water control and oil enhancement” development feature and the best step-wise oil production effect. The oil recovery rate of the two schemes for fine conglomerate shows a difference of 10.14%, followed by 6.36% for glutenite and 5.10% for sandstone. In addition, the EOR of fine conglomerate maintains a high upward trend throughout the chemical flooding, indicating that the swept volume of small pore throats gradually expands and the producing degree of the remaining oil in it gradually increases. Therefore, the fine conglomerate is the most suitable lithology for the SCV scheme among the three lithologies of the conglomerate reservoirs.

## 1. Introduction

A binary flooding system composed of polymer and surfactant is an effective recovery enhancement agent after water flooding development in high water-content oil fields [1]. Overall, the permeability of reservoirs is decreased due to the adsorption and retention of clay minerals on surfactant–polymer (SP) components [2]. The effect of enhancing oil recovery mainly depends on the interaction mechanism within the reservoir [3]. When the SP binary solution enters the porous medium, on the one hand, it expands the sweep volume of the binary system by increasing the viscosity of the agent and decreasing the oil–water mobility ratio [4]; on the other hand, it increases the oil displacement efficiency by changing the wettability of the rock and decreasing the adsorption capacity of the remaining oil [5]. The synergistic effect of the two approaches can significantly improve the oil displacement effect and enhance reservoir recovery [1,6]. At present, scholars at home and abroad have conducted a lot of work in the field of SP binary flooding, mainly including the interaction mechanisms of binary systems [7,8,9], the study of interfacial rheology [10,11], the optimization of injection parameters [12,13], and the modification of polymer rheological properties by nanoparticles [14,15,16], which effectively reveal the oil displacement mechanism of SP binary system, determine the optimal parameters of SP binary flooding in different reservoirs, and achieve good development results in field applications [17,18,19]. However, the above research results are basically from single-mode sandstone reservoirs, and there are relatively few studies for complex mode conglomerate reservoirs due to their relatively limited global distribution, special depositional environment, and complex pore structure. 

Due to the special depositional environment of conglomerate reservoirs, the microscopic pore structure of the reservoir shows complex mode characteristics, with extremely uneven pore throat distribution, poor connectivity, and strong anisotropy [20,21,22,23]. The complex pore structure controls the seepage characteristics and production rules of crude oil in different scale pore throats [24]. For the oil displacement process of different media, the microscopic manifestation is mainly the large difference in fluid flow patterns in different cores, which is reflected in the macroscopic phenomenon as the obvious difference in development characteristics, which in turn leads to significant distinctions in the final recovery rates of different types of reservoirs in conglomerate reservoirs [25]. In addition, when the SP binary system enters the pore medium, it will have various physicochemical reactions with rock particles and crude oil. Different lithologies have different depositional environments and diagenesis, and the interfacial rheological properties and pore structure of sedimentary particles vary greatly, resulting in different production degrees of crude oil in different scales of pore throats, which, in turn, affects the enhanced oil recovery (EOR) of different lithologies by SP binary flooding [26]. Therefore, in order to realize the purpose of EOR in conglomerate reservoirs, it is necessary to conduct an in-depth study on the displacement process of different lithologies according to their macro and micro characteristics, combined with the reservoir geology and development dynamic data, to clarify the difference in oil displacement rules by SP binary flooding to formulate a more efficient development plan and to provide a theoretical basis for the high and stable production of oil fields. 

At present, to further improve the oil recovery of conglomerate reservoirs, some scholars have applied the theory of “echelon injection and step-wise production” to compare and analyze the effect of the echelon viscosity increasing (EVI) scheme, single constant viscosity (SCV) scheme, and echelon viscosity reducing (EVR) scheme [27]. Among these, the EVR scheme realizes the reconstruction of the pressure field in porous media and the redistribution of seepage paths of different size chemical micelles by reducing polymer molecular weight and solution viscosity during the displacement process, so as to improve the oil displacement efficiency of large pore throats and expand the sweep volume of small pore throats, to increase the oil production degree of different scale pore throats, and, finally, to achieve the purpose of further improving the recovery rate of conglomerate reservoirs [28,29]. Tan et al. [30] applied this method to the K7 block of the conglomerate reservoir in the Karamay Oilfield and solved the problem of low oil production and high water content during the production, which achieved good results. However, all current studies have neglected considering the impact of reservoir anisotropy on step-wise production schemes, i.e., the variability in oil production rules in conglomerate reservoirs with different pore structures during SP binary displacement, which is a key factor affecting the overall EOR. In this paper, full-diameter natural cores from the conglomerate reservoir of Kexia Formation in the seventh middle block of the Karamay Oilfield (Xinjiang, China) are used to conduct displacement experiments to study the influence of different lithologies on the EOR by EVR scheme, to clarify the variability of oil displacement rules, and to provide a geological basis for the overall design of the reservoir chemical displacement scheme. 

## 2. Geological Background

**Figure 1:**
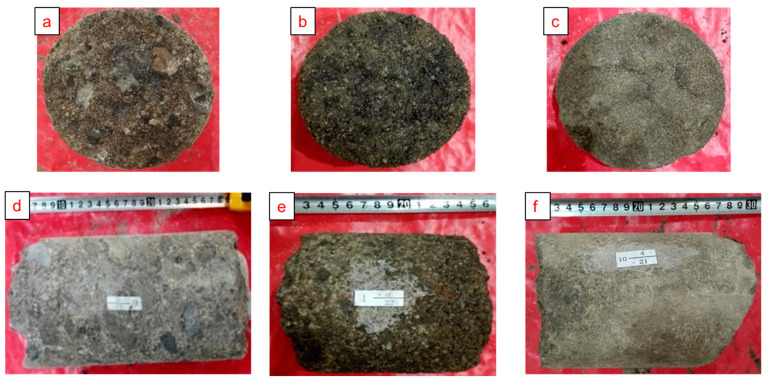
Core photos of typical lithologies; (**a**) transverse core photo of fine conglomerate; (**b**) transverse core photo of glutenite; (**c**) transverse core photo of sandstone; (**d**) longitudinal core photo of fine conglomerate; (**e**) longitudinal core photo of glutenite; (**f**) longitudinal core photo of sandstone.

**Figure 2:**
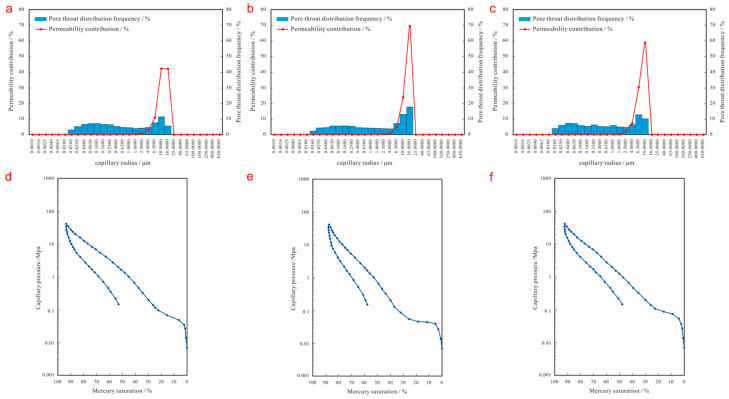
Microscopic pore structure characteristics of typical lithologies; (**a**) pore throat distribution of fine conglomerate; (**b**) pore throat distribution of glutenite; (**c**) pore throat distribution of sandstone; (**d**) intrusive mercury curve of fine conglomerate; (**e**) intrusive mercury curve of glutenite; (**f**) intrusive mercury curve of sandstone.

Located at the northwest edge of Junggar Basin, the Karamay Oilfield is one of the most important oil and gas production centers in China, and also the distribution area of conglomerate reservoirs with some of the largest reserves in the world. Due to the near-source, multi-stream system and fast-changing sedimentary environment, the conglomerate reservoir as a whole is a set of alluvial-fan sediment and, through the modification of late diagenesis, the microscopic pore structure shows the characteristics of complex mode distribution [31], which belongs to the strong anisotropy reservoir [32]. The Lower Karamay Formation of Triassic, referred to as the Kexia Formation, is one of the main oil-bearing formations in the Junggar Basin. The Kexia Formation is composed of three sand groups: S8, S7, and S6, with a reservoir thickness of up to 130 m [33]. The oil-bearing area of the conglomerate reservoir of the Kexia Formation in the seventh middle block is 0.44 km^2^, with an average oil thickness of 14.6 m, and a crude oil reserve of 54 × 10^4^ t [34]. The main applicable formations of SP binary flooding are S_7_^2–2^~S_7_^4–1^, and the main oil-bearing lithologies are fine conglomerate, glutenite, and sandstone (Figure 1). Due to the variability of sedimentary environment, hydrodynamic conditions, and late diagenesis, different lithologies have different microscopic pore structure characteristics (Figure 2). Among them, the fine conglomerate sedimentary particles are the worst sorted, with low structural maturity, extremely uneven pore throat distribution, frequency histograms with bimodal fine-shaped characteristics, extensive development of conglomerate gravel edge fractures and intragranular fractures, and more small–medium throat channels; the glutenite sedimentary particles are poorly sorted, with low structural maturity, more uniform pore throat distribution, frequency histograms with single-peaked coarse-shaped characteristics, more medium throat channels, and average pore throat connectivity; the sandstone sedimentary particles are better sorted, with high structural maturity, uniform pore throat distribution, frequency histograms with single-peaked fine-shaped characteristics, and good pore throat connectivity.

Since 2018, the conglomerate reservoirs of Kexia Formation in the seventh middle block have been continuously optimized for the SP binary flooding scheme, which has achieved good application results with outstanding economic benefits, and the relevant technical achievements have a wide range of prospects for popularization and application. However, some pressing problems have been identified in the field tests, among which the effect of anisotropy of conglomerate reservoirs on the production rules and oil recovery rate is one of the most important issues. Different lithologies have different microscopic pore structure characteristics. If a uniform SP binary flooding scheme is used in the reservoir, the applicability of the chemical agent becomes worse, which in turn affects the overall development effect of the reservoir. At the same time this will lead to some production dynamic problems, such as obvious emulsion output, high chemical agent output concentration, a significant decrease in fluid production capacity, and uneven formation pressure. Eventually, it will cause a limited SP flooding sweep volume and a serious channeling phenomenon. Therefore, addressing the influence of anisotropy on the recovery rate of SP binary flooding in conglomerate reservoirs and clarifying the variability of oil production rules in different lithologies are the keys to efficient development. 

## 3. Experimental Design

### 3.1. Experimental Materials and Equipment

The experimental cores are three full-diameter natural cores from the binary test area of the Kexia Formation in the seventh middle block, and cores of three lithologies, including fine conglomerate, glutenite, and sandstone, were selected to simulate the process of the step-wise production of crude oil in the pore throat, and the parameters (single values) of each core are shown in Table 1.

The experimental water was simulated formation water from the test area, and its ionic composition is shown in Table 2. The oil used for the experiment is the simulated oil after 1:2 compounding of crude oil and kerosene extracted from the Kexia Formation in the seventh middle block. In the SP binary complex flooding chemical system, polyacrylamide (HPAM) was used for the polymer, and petroleum sulfonate (KPS202) was used for the surfactant.

The experimental equipment included: (1) Waring stirrer; (2) electronic balance: JA2003A with the accuracy of 1 mg; (3) electronic balance: ES-10K-4TS type with the accuracy of 0.1 g; (4) interfacial tension meter; (5) Brinell viscometer; (6) FY-3 thermostat; (7) ISCO repellent pump; (8) full-diameter core gripper; (10) pressure acquisition equipment.

### 3.2. Experimental Schemes

For natural core samples with different lithologies in conglomerate reservoirs, two schemes of SCV and EVR are designed to displace oil. For the SCV scheme, the agent concentration and polymer relative molecular mass are kept constant, while the EVR scheme reduces the solution viscosity by changing the agent concentration and polymer relative molecular mass to achieve effective displacement of crude oil in different scale pore throats and to attain the purpose of step-wise production. The specific parameters of the oil displacement schemes are shown in Table 3.

### 3.3. Experimental Steps

**Figure 3:**
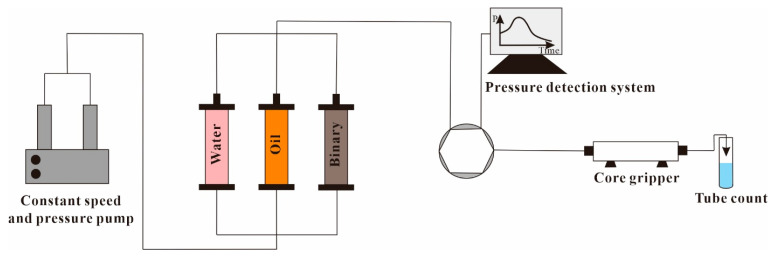
Experimental flow chart.

In order to make the experimental results more consistent with the real reservoir displacement process, the experiments were conducted under the reservoir temperature (42 °C) and formation pressure (16.2 MPa) conditions, and the specific processes were as follows: (1) the basic data, such as diameter, length, and dry weight of three full-diameter natural cores were measured; (2) the rock samples were vacuumed and saturated with water, and then displace with the configured simulated oil to establish the bound water model, and calculate the porosity of the rock sample; (3) we connected the experimental process shown in Figure 3, adjusted various instrument parameters, and started the oil displacement test; (4) in water flooding process, the rock sample was displaced with simulated formation water to above 95% water content to stop the experiment, and we recorded the experimental data, such as oil production and pressure change; (5) in the process of SP binary oil displacement, on the basis of water flooding, two displacement schemes, SCV and EVR, were used to chemically displace the three rock samples at a displacement rate of 0.6 mL/min, the experiment was stopped until the water content at the outlet reached more than 95%, and the experimental data, such as oil production and pressure changes, were recorded; (6) the rock samples were subsequently water-flooded and the data were recorded and processed (Figure 3). 

## 4. Results and Discussion

### 4.1. Step-Wise Oil Production Rule of Fine Conglomerate

**Figure 4:**
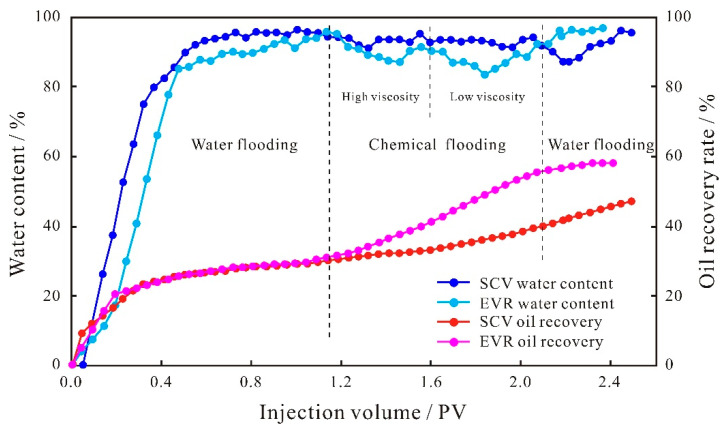
Production characteristic curves of small conglomerate.

As can be seen from the production characteristic curves of fine conglomerates (Figure 4), for the initial water flooding stage, the oil recovery rate curves basically overlap during the two displacements, and the oil recovery rate values remain the same, because the properties of the injection water and the pore structure of the core samples do not change; although the water content values have small differences, the curves are basically the same. Due to the strong anisotropy of the fine conglomerate, the connectivity between the pore throats is poor, and the sweep volume of injected water is limited, resulting in a relatively low water flooding recovery rate of 29.91% on average. After water flooding, two chemical displacement schemes were performed on the cores separately; when the SP binary system entered the microscopic pore medium, the polymer could effectively increase the viscosity of the displacement fluid and expand the sweep volume of the binary system by changing the oil–water mobility ratio [35,36]. Additionally, surfactants can change the wettability of the rock and improve the oil displacement efficiency of crude oil by reducing the oil–water interfacial tension [37,38], and the two approaches work synergistically to improve the reservoir recovery. Based on the above mechanism, the SCV scheme can effectively improve the oil recovery rate, and with the continuous injection of the SP binary system, the recovery rate curve shows a linear increasing trend, and the water content remains stable after the reduction, forming the development characteristic of “water control and oil enhancement “. By the end of the SP binary flooding, the oil recovery reached 48.63%, which is an 18.72% increase in recovery compared to the water flooding.

Compared with the SCV scheme, the overall development effect of the EVR scheme is better. Firstly, the oil recovery degree showed an exponential increase, forming a large difference from that of the SCV scheme. By the end of the binary flooding, the oil recovery rate reached 58.54%, which was 28.63% higher compared with the water flooding, and 9.91% higher compared with the SCV scheme; Secondly, the overall water content is lower than that of SCV scheme, with a decrease of 10.59%, indicating that the EVR scheme has a better water control effect. In addition, there are also differences in the development effects of the two displacement stages of the EVR scheme. When the high-index, highly concentrated, strongly emulsified agent enters the microscopic pore space of the reservoir, it will form a high resistance area in the large pore throat on the mainstream line of the water flooding, lowering the flow of the displacement fluid in the dominant seepage channel, reducing water channeling and improving the oil displacement efficiency of the large pore throats, which has a limited contribution to the recovery enhancement due to the small amount of remaining oil in the large pore throats. As a result, the EOR at this stage is smaller and the reduction in water content is lower. When the molecular weight and viscosity are lowered, the low molecular weight and low-density agent mainly produce the low-permeability layer. By the reconstruction of the pressure field and the redistribution of seepage paths of chemical micelles with different sizes, the oil production rule shows a trend of shifting from large pore throats to small pore throats. The lower production limit is greatly reduced after the viscosity reduction, which expands the sweep volume of small pore throats and realizes the step-wise oil production of different scales of pore throats. Since the remaining oil in the small pore throats was largely basically un-replaced, the contribution degree of recovery was greater. Therefore, the magnitude of the increase in recovery rate and the reduction in water content are both more significant in the low-viscosity stage (Figure 4).

**Figure 5:**
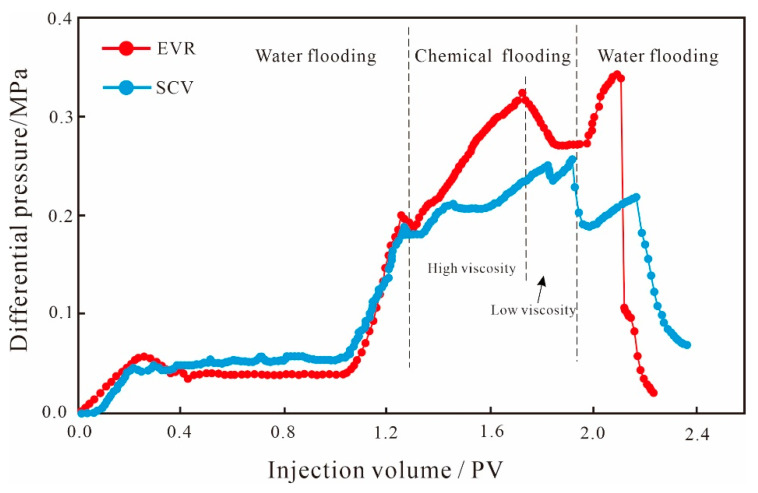
Injection pressure difference curves of small conglomerate.

The injection pressure difference between the two ends of the core samples during oil displacement can reflect the seepage capacity at different stages. The increase in the injection pressure difference indicates that the seepage resistance increases, the seepage capacity of the dominant channel decreases, and the injected solution will flow toward the smaller pore space, which is conducive to the displacement of the remaining oil in the small pore throats, improving the recovery rate of the reservoir and reducing the water content. From the injection pressure difference curves of the small conglomerate sample (Figure 5), it can be seen that the pressure curves of the two schemes of SCV and EVR in the water flooding stage remain basically the same, indicating that the seepage capacity of the solution does not change and the oil production degree in the pore throat remains the same. When entering the SP binary flooding stage, for the SCV scheme, the injection pressure difference shows a continuous increase because the large molecular weight polymer will form a high resistance area in the large pore throats on the mainstream of the water flooding, reducing the flow of the agent in the dominant percolation channel and increasing the percolation resistance, causing the binary system to percolate more into the small pore throats, which in turn requires overcoming a larger capillary force. As for the EVR scheme, due to the fact that that the concentration and viscosity of the binary solution in the high-viscosity stage are higher than that of the SCV scheme, when the binary system enters the microscopic pore structure, it will form a higher resistance area in the large pore throats in the mainstream line of water flooding, and the seepage capacity of the dominant channel is reduced to a larger extent; therefore, the injection pressure difference is higher than that of the SCV scheme, and shows a substantial increase in trend. When the molecular weight and viscosity are reduced, the injection pressure difference gradually decreases through the pressure field reconstruction and the seepage paths redistribution, and the low molecular weight and low-density agent can flow toward the large pore throats as well as the small–medium pore throats where seepage channels have been formed. However, for the SP binary flooding stage, the overall injection pressure difference in the EVR scheme is higher than that of the SCV scheme. 

### 4.2. Step-Wise Oil Production Rule of Glutenite

**Figure 6:**
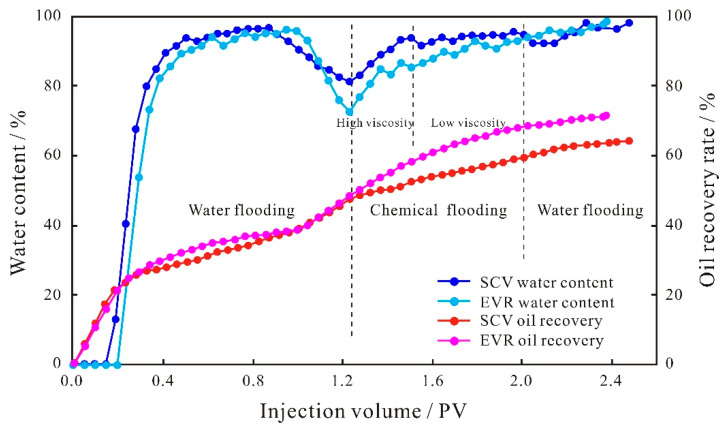
Production characteristic curves of glutenite.

As can be seen from the production characteristic curves of glutenite (Figure 6), for the initial water flooding stage, the oil recovery rate curves basically overlap with little change in values because the properties of the injection water and the pore structure of the core samples do not change; the water content values have small differences, but the curve shapes are basically the same. As the anisotropy of the glutenite is weaker than that of the fine conglomerate, the pore throat connectivity is better, which leads to the increase in the swept volume of the injected water; the average oil recovery rate of water flooding is 37.40%, which is 7.49% higher than that of fine conglomerate, indicating that the reservoir anisotropy has a greater influence on the water flooding development effect of the conglomerate reservoir. After water flooding, two chemical displacement schemes were separately performed on the cores. From the production characteristic curves, it can be seen that the SCV scheme can effectively increase the oil production degree, the oil recovery rate curve shows a linear increasing trend with the continuous injection of the SP system, and the water content shows a trend of first decreasing and then increasing; finally, it remains stable. By the end of the SP binary flooding, the oil recovery rate reached 64.25%, which was 27.12% higher compared to the water flooding, while the chemical displacement recovery was 8.85% higher compared to the anisotropy fine conglomerate, indicating that reservoir anisotropy also has an impact on the development effect of the SP binary flooding.

Compared with the SCV scheme, the overall development effect of the EVR scheme is better. Firstly, the oil recovery degree showed an exponential increase: by the end of binary flooding, the oil recovery rate reached 71.29%, which was 33.89% higher compared with the water flooding, and 6.77% higher compared with the SCV scheme. Secondly, the overall water content is lower than that of SCV scheme, with a decrease of 8.59%, indicating that the EVR scheme has a better water control effect. In addition, there are also differences in the development effects of the two displacement stages of the EVR scheme. During the high-viscosity stage, the degree of oil recovery increases to a lesser extent, and the water content shows a gradually increasing trend. When the molecular weight and viscosity are reduced, the reconstruction of the pressure field stabilizes the seepage paths of the binary solution, and the water content remains unchanged, while the binary system with low molecular weight and low concentration enters more into the smaller pore space, expanding the sweep volume of the small pore throats. As such, the oil recovery rate shows a substantial increase and the development effect becomes better (Figure 6).

**Figure 7:**
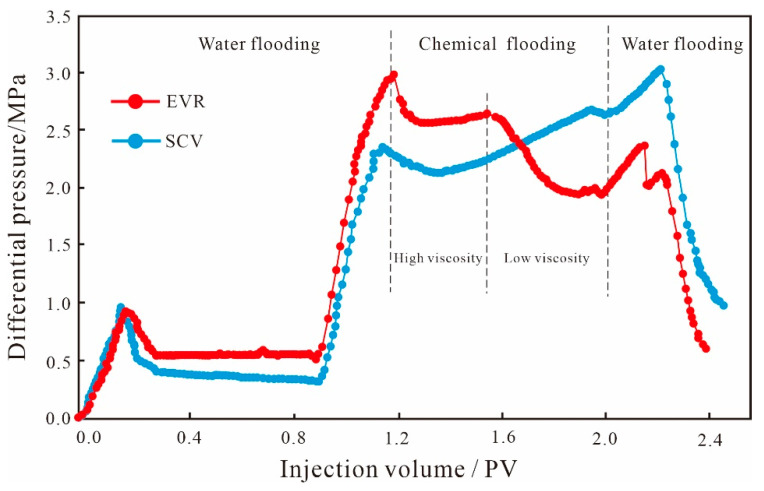
Injection pressure difference curves of glutenite.

It can be seen from the injection pressure difference curves between the two schemes of glutenite (Figure 7) that the curves of the SCV and EVR schemes in the water flooding stage remain basically the same, indicating that the seepage capacity of the solution does not change, and the oil production degree remains unchanged. When entering the SP binary flooding stage, for the SCV scheme, the injection pressure difference shows a continuous upward trend due to the formation of a high resistance area on the dominant seepage channel by the large molecular weight polymer. For the EVR scheme, because the concentration and viscosity of the binary solution in the high molecular weight and high concentration stage are higher than that of the SCV scheme when the binary system enters the microscopic pore space, it will form a higher resistance area in the large pore throats on the mainstream line of water flooding. Therefore, the injection pressure difference is higher than that of the SCV scheme and shows a gradual increase. When the molecular weight and viscosity are reduced, the low molecular weight and low concentration binary system can not only flow in large pore throats but can also enter into smaller pore spaces through the reconstruction of the pressure field and the redistribution of seepage paths of chemical micelles with different sizes, and the injection pressure difference is gradually reduced. Due to the weak anisotropy and better pore throat connectivity of glutenite, the SP binary solution of low molecular weight and low concentration that enters the small–medium pore throats has less capillary force to overcome, resulting in a smaller injection pressure difference than the SCV scheme at this stage, unlike the variation characteristics of the fine conglomerate. It is fundamentally determined by the microscopic pore structure of the reservoir. 

### 4.3. Step-Wise Oil Production Rule of Sandstone

**Figure 8:**
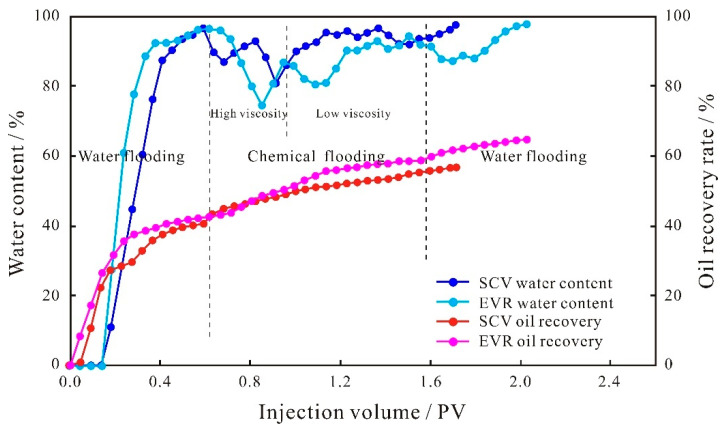
Production characteristic curves of sandstone.

The production characteristic curves of the initial water flooding stage of the sandstone are the same as the other two types of lithologies (Figure 8), and the oil recovery rate of water flooding and water content curves have basically the same shape with small differences in values. The sandstone has the weakest anisotropy and better pore throat connectivity than fine conglomerate and glutenite, resulting in the largest sweep volume of injected water among the three types of lithologies. Its average oil recovery rate of water flooding reaches 41.83%, which is 11.92% and 4.43% higher than that of fine conglomerate and glutenite, respectively, indicating that reservoir anisotropy has a relatively large impact on the water flooding development effect of conglomerate reservoirs. After the end of water flooding, the cores were displaced by two chemical displacement schemes separately. From the production characteristic curves, it can be seen that the SCV scheme can effectively increase the oil production degree, the oil recovery rate curve shows a linear increasing trend with the continuous injection of the SP system, and the water content shows a decreasing trend. By the end of the SP binary flooding, the oil recovery rate reached 58.12%, which was 16.29% higher compared to the water flooding. Compared with the fine conglomerate and glutenite, the chemical oil recovery rates are reduced by 2.43% and 10.83%, respectively. On the one hand, due to the relatively high water flooding oil recovery rate of the sandstone reservoir, the magnitude of chemical flooding to enhance the recovery rate is limited; on the other hand, it indicates that the SCV scheme is not well-matched with the pore structure of the sandstone reservoir. 

Compared with SCV scheme, the overall development effect of the EVR scheme is better. By the end of the binary flooding, the oil recovery rate reached 64.81%, which was 22.98% higher compared with the water flooding, and 6.69% higher compared with the SCV scheme. The water content is also lower overall than that of the SCV scheme, with a decrease of 7.39%, indicating that the EVR scheme has a better water control effect. In addition, there are also differences in the development effects of the two displacement stages of the EVR scheme. During the high-viscosity stage, the degree of oil recovery increases to a relatively small extent and the water content shows a decreasing trend. When the molecular weight and viscosity are reduced, the reconstruction of the pressure field stabilizes the seepage paths of the binary solution, the water content remains unchanged, and the binary system with low molecular weight and low concentration enters more into the smaller pore space, expanding the sweep volume of small pore throats; as such, the oil recovery rate shows a substantial increase. The water content first decreases and then gradually increases, and finally remains stable, and the development effect becomes more effective (Figure 8).

**Figure 9:**
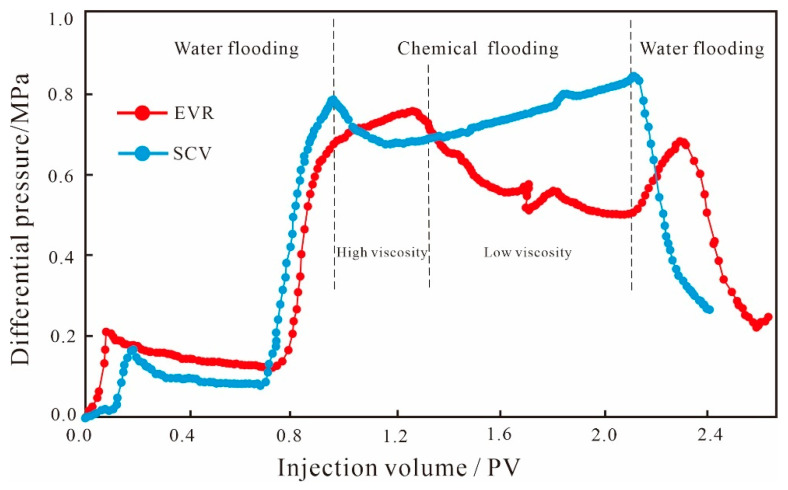
Injection pressure difference curves of sandstone.

The injection pressure difference curve in the water flooding stage of sandstone is similar to the other two types of lithologies (Figure 9), and the curves of the SCV and EVR schemes remain basically the same, indicating that the seepage capacity of the solution and the oil production degree remain unchanged. When entering the SP binary flooding stage, for the SCV scheme, the injection pressure difference shows a continuous upward trend due to the formation of a high resistance area on the dominant seepage channel by the large molecular weight polymer. For the EVR scheme, since the concentration and viscosity of the binary solution in the high-viscosity stage are higher than that of the SCV scheme, it will form a higher resistance area in the large pore throats on the mainstream line of water flooding when the binary system enters the microscopic pore space. As a result, the EVR scheme has a higher injection pressure difference and shows a small upward trend. When the molecular weight and viscosity are reduced, the low-viscosity agent can not only flow in large pore throats, but also enter into smaller pore spaces by the reconstruction of the pressure field and the redistribution of seepage paths. Since the anisotropy of sandstone is the weakest among the three types of lithologies, the pore throat connectivity is better than the other two types of lithologies, and the low-viscosity SP binary solution has the least capillary force to overcome to enter the small–medium pore throats with the lowest seepage resistance. Therefore, the injection pressure difference at this stage is lower than that of the SCV scheme and shows a substantial decrease in variation. 

### 4.4. Difference in Step-Wise Production Rules of Various Lithologies

#### 4.4.1. Difference in Oil Recovery Rates

For different lithological types of conglomerate reservoirs, when the parameters and scheme of the SP binary system are determined, the main factor affecting the recovery rate is the properties of the reservoir itself, including lithology, physical properties, pore structure, clay content, etc. A comparative analysis of the enhanced recovery rates of the two chemical displacement schemes of SCV and EVR for the three types of lithologies shows that the EOR of the different lithologies varies greatly, and the EOR of the SCV and EVR schemes for glutenite are the largest, reaching 27.19% and 33.55%, respectively, followed by fine conglomerate and sandstone. In addition, for the same lithology, the EOR between the SCV and EVR schemes also differed significantly, with the largest difference of 10.14% for fine conglomerate, followed by 6.36% for glutenite, with the smallest being 5.10% for sandstone (Table 4). 

**Figure 10:**
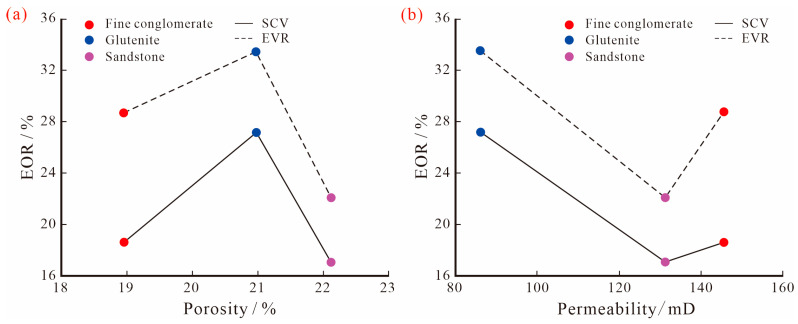
(**a**) The relationship between porosity and EOR; (**b**) The relationship between permeability and EOR.

In order to clarify the controlling factors of the oil production degree during SP binary flooding in conglomerate reservoirs, the correlation between physical parameters and EOR of different lithologies was analyzed. For both the SCV and EVR schemes, there is no significant linear relationship between the EOR and both porosity and permeability (Figure 10), indicating that reservoir physical properties are not the main controlling factor for recovery enhancement by chemical displacement, which is different from the conventional SP production rule for sandstone reservoirs. The main reason is that, due to the special sedimentary environment and complex late diagenesis of conglomerate reservoirs, the pore throat structure is characterized by a “complex mode”, referring to poor pore throat connectivity, small mean throat radius, and high interstitial content, meaning that the macroscopic physical parameters cannot truly reflect the microscopic seepage rules. This results in a poor correlation between the reservoir’s physical properties and EOR by SP binary displacement.

**Figure 11:**
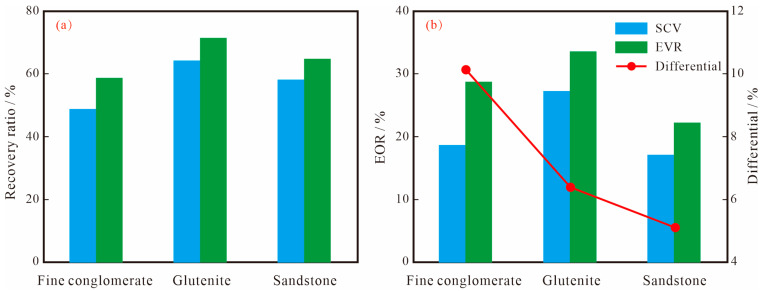
Relationships between lithology and oil recovery; (**a**) the relationship between lithology and recovery ratio; (**b**) the relationship between lithology and EOR.

For conglomerate reservoirs with strong anisotropy, there are large differences in the pore structure of different lithologies, resulting in different microscopic seepage paths and the production rules of SP binary solutions, as well as large differences in EOR. Among them, the two schemes on glutenite have the largest EOR, with 27.19% and 33.55%, respectively, followed by fine conglomerate with 18.62% and 28.76%, respectively, while sandstone is the smallest with 17.09% and 22.19%, respectively. Therefore, the glutenite reservoir is a potential layer for SP binary composite displacement. In addition, the comparison of the recovery rates of the three lithologies shows that the recovery rates of EVR are higher than those of SCV, which indicates that the EVR scheme can make the oil displacement system of low molecular weight and low concentration not only flow effectively in large pore throats, but also enter into smaller pore spaces to displace crude oil, and increase the production degree of remaining oil by improving the displacement efficiency and expanding the swept volume, thus, enhancing the oil recovery rate. However, due to the large differences in the microscopic pore structure of the three lithologies, the difference in the EOR between the two schemes is not the same. Overall, the difference in fine conglomerate is greater than that of glutenite and sandstone (Figure 11), indicating that the stronger the anisotropy and the poorer the pore throat connectivity of the reservoir, the better the effect of step-wise production, and the EVR scheme is more favorable to improve the recovery of this type of reservoir. 

#### 4.4.2. Difference in Seepage Characteristics

**Figure 12:**
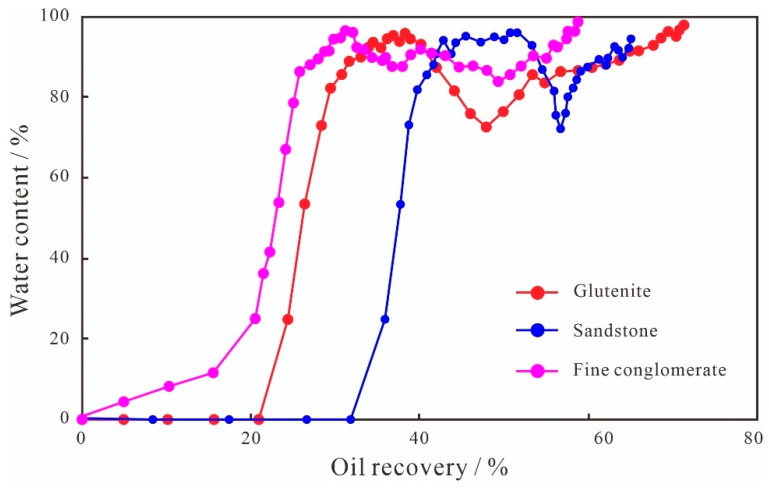
Production characteristic curves of fine conglomerate, glutenite, and sandstone.

The microscopic pore structure of the three types of lithologies differs greatly. For the EVR scheme, the production characteristic curves of different displacement stages show different trends. From the variation curve of water content (Figure 12), it can be seen that both sandstone and glutenite have a water-free oil production period, indicating that the seepage characteristics of sandstone and glutenite are similar. The length of the water-free oil production period is related to the anisotropy degree and pore structure of different lithologies. The stronger the anisotropy of the reservoir, the poorer the pore throat connectivity, and the easier it is to form dominant seepage channels. When the injected water enters the microscopic pore medium, the capillary resistance in the dominant seepage channel is easily broken, which leads to the water channeling, resulting in a shorter water-free oil production period and poorer water displacement efficiency. Therefore, the production degree during the water-free oil production period is 21.43% for the glutenite and 32.76% for the sandstone, and the degree of water-free production for the sandstone is 11.33% higher than that for the glutenite. The fine conglomerate has the strongest anisotropy among the three types of lithologies, the dominant seepage channel is developed, and water channeling is serious, so the fine conglomerate basically has no water-free oil production period, and the water displacement effect is the worst.

**Figure 13:**
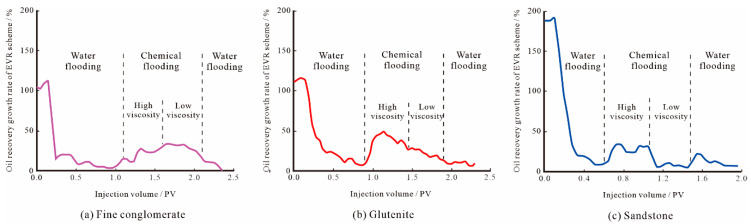
Oil recovery growth rate of the EVR scheme for different lithologies.

The oil recovery growth rate of the EVR scheme at each stage can be obtained by differentiating the oil recovery rate curve in the EVR scheme (Figure 13). For the water flooding stage, the oil recovery rate of sandstone grows fastest, followed by the glutenite, and the fine conglomerate grows slowest, which is generally consistent with the trend of the production characteristic curves above. As for chemical flooding stage, when the high molecular weight and high-viscosity oil displacement system enters the porous medium, it will firstly form a high resistance area in the large pore throat seepage channel, which will significantly lower the flow degree of the water and reduce the water channeling. After that, the low molecular weight and low-viscosity binary system will be injected, which will increase the sweep volume in the low flow degree seepage channel formed by the high-viscosity polymer and improve the production degree of the remaining oil in the small pore throats. As a result, the oil recovery rate increased in all of the EVR schemes, but there were differences in the magnitude of the increase.

The increase in the injection multiple reflects, to some extent, the gradual seepage of the binary system from large pore throats to small pore throats, and the corresponding oil recovery growth rate also reflects the production degree of crude oil in different scales of pore throat. The oil recovery growth rate of fine conglomerate remains high throughout the chemical flooding, with the low-viscosity stage slightly higher than the high-viscosity stage, indicating the most effective step-wise production within different scale pore throats of fine conglomerate. The oil recovery growth rate of sandstone is faster in the high-viscosity stage and slower in the low-viscosity stage, mainly because the polymer molecules in the SP binary composite system can fit into most of the pore throats in the reservoir during the chemical flooding stage, but cannot enter the smaller pore throats for effective displacement, resulting in a limited sweep volume of crude oil in the small pore throats and a low recovery rate. From the sweep volume and oil production degree in the different scales of pore throats, it can be seen that the sweep volume of fine conglomerate is the largest, its oil production degree is the highest, and its step-wise production effect is the best. Therefore, the fine conglomerate is the most suitable reservoir type for the EVR scheme among the three lithologies, the glutenite is the second best, and the step-wise production effect of sandstone is the worst.

#### 4.4.3. Difference in Injection Pressure Difference

**Figure 14:**
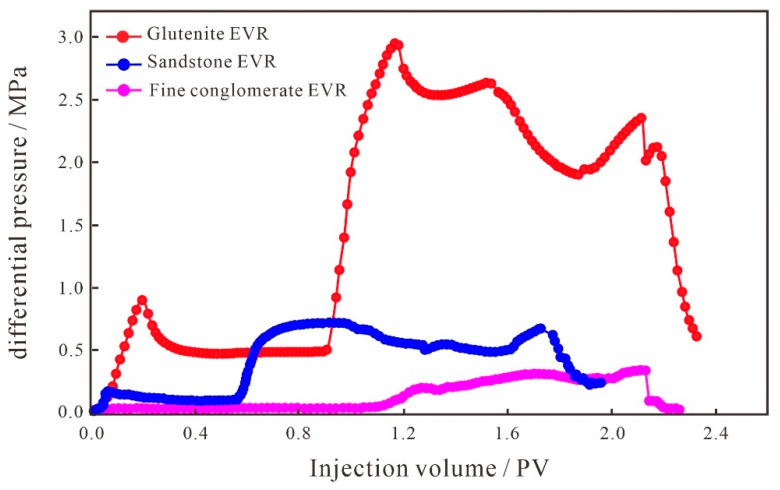
The relationship between different lithologies injecting differential pressure curves.

From the relationship between injection pressure difference and multiple injections for the different lithologies of the EVR scheme (Figure 14), it can be seen that in the initial water flooding stage, the pressure difference in glutenite is the highest, followed by sandstone, and the fine conglomerate has the smallest pressure difference; when entering the chemical flooding stage of the SP binary system, the injection pressure difference in glutenite is significantly higher than that of fine conglomerate and sandstone. The underlying reason is that the permeability of glutenite is significantly lower than the other two types of lithologies, with a complex pore structure and poor pore throat connectivity, and the capillary resistance to be overcome by the displacement medium is relatively large, so the injection pressure difference is the largest among the three types of lithologies. Although the fine conglomerate shows more anisotropy and poorer pore throat connectivity than the glutenite, the development of dominant seepage channels in the microscopic pores of the fine conglomerate results in a smaller injection pressure difference than the glutenite. The permeabilities of fine conglomerate and sandstone are similar, but the difference in pressure is still large, mainly due to the strong anisotropy of fine conglomerate, poor sorting and rounding of sedimentary particles, low structural maturity, weak diagenesis, and the development of gravel edge fractures or microfractures, the existence of which will lead to easy channeling during the displacement process. In addition, the development of the dominant seepage channel will also reduce the seepage resistance of the displacement solution, which will form the water channeling and eventually lead to the minimum pressure difference in the fine conglomerate. In summary, combined with the EVR scheme, the mechanism of a “high-viscosity agent reduces water channeling and low-viscosity agent increases sweep volume” can well explain the step-wise production effect in conglomerate reservoirs, with fine conglomerate being the best, glutenite the second best, and sandstone the worst.

## 5. Conclusions

The following conclusions were obtained by using two different experimental schemes to simulate the crude oil production process for three cores from the Triassic Kexia Formation in the Junggar Basin.

(1) By reducing the molecular weight and viscosity of the SP binary system, the EVR scheme can realize reconstruction of the pressure field and the redistribution of seepage paths of chemical micelles with different sizes, which can make the low molecular weight and low concentration agent not only flow effectively in large pore throats, but also allows seepage into smaller pore spaces to displace crude oil, thus, improving the oil recovery of the reservoir.
(2) The EVR scheme can achieve the step-wise production of crude oil in different scales of pore throats in conglomerate reservoirs, and the main factor affecting the step-wise production effect is the microscopic pore structure characteristics. The fine conglomerate reservoir has the strongest anisotropy and the worst pore throat connectivity, but the swept volume of small pore throats after viscosity reduction is the largest, and the EOR difference in the two schemes is also the largest, reaching 10.14%. The step-wise oil production effect of fine conglomerate is the best, followed by glutenite and sandstone.
(3) The oil recovery growth rate curve can well reflect the gradual sweep of the SP binary system from large pore throats to small pore throats in the EVR scheme. The oil recovery growth rate of fine conglomerate maintains a high increasing trend throughout the chemical flooding process, while the growth rate of glutenite and sandstone shows a gradual decreasing trend, indicating that fine conglomerate has the best step-wise oil production effect in different scale pore throats and is the most suitable reservoir type for the EVR scheme among the three types of lithologies.

In conclusion, this study determined the influence of lithology on the enhanced oil recovery of the EVR scheme, which in turn revealed the differences in the step-wise production rules of three lithologies, thus, ensuring the efficient application of SP binary flooding in the development of conglomerate reservoirs.

## Figures and Tables

**Table 1:** Parameters of experimental cores.

Lithology	Fine Conglomerate	Glutenite	Sandstone
Length/cm	17.70	12.60	15.60
Diameter/cm	11.12	10.89	11.96
Porosity/%	18.96	20.98	22.12
Gas Permeability/mD	145.60	86.20	131.20
Oil saturation/%	55.31	52.16	67.02

**Table 2:** Ionic composition of formation water.

Ionic Type	Na^+^	K^+^	Ca^2+^	Mg^2+^	CO_3_^2−^	HCO_3_^−^	SO_4_^2−^	Cl^−^	Total Mineralization
Concentration mg/L	1595.60	1085.70	18.00	9.46	109.79	1452.50	40.57	2500	6811.62

**Table 3:** Experimental parameters of the oil displacement schemes.

Lithology	Scheme	Oil Displacement System
Fine Conglomerate	SCV	1900 × 10^4^ 1200 mg/L HPAM, 0.25% KSP202
	EVR	1900 × 10^4^ 1500 mg/L HPAM, 0.3% KSP202 + 1900 × 10^4^ 900 mg/L HPAM, 0.2% KSP202
Glutenite	SCV	1900 × 10^4^ 1200 mg/L HPAM, 0.25% KSP202
	EVR	1900 × 10^4^ 1500 mg/L HPAM, 0.3% KSP202 + 1900 × 10^4^ 900 mg/L HPAM, 0.2% KSP202
Sandstone	SCV	1900 × 10^4^ 1200 mg/L HPAM, 0.25% KSP202
	EVR	1900 × 10^4^ 1500 mg/L HPAM, 0.3% KSP202 + 1900 × 10^4^ 900 mg/L HPAM, 0.2% KSP202

**Table 4:** Recovery rates of different lithologies.

Lithology	Gas Permeability/mD	Porosity/%	Initial Oil Saturation/%	Scheme	Oil Recovery Rate/%			
					Water Flooding	Chemical Flooding	EOR	Difference
Fine conglomerate	145.6	18.96	55.31	SCV	30.01	48.63	18.62	+10.14
				EVR	29.78	58.54	28.76	
Glutenite	86.2	20.98	52.16	SCV	37.06	64.25	27.19	+6.36
				EVR	37.74	71.29	33.55	
Sandstone	131.2	22.12	67.02	SCV	41.03	58.12	17.09	+5.10
				EVR	42.62	64.81	22.19	

## Data Availability

The data presented in this study are available on request from the corresponding author. The data are not publicly available due to the need for further relevant re-search.
